# MiRNA-related metastasis in oral cancer: moving and shaking

**DOI:** 10.1186/s12935-023-03022-5

**Published:** 2023-08-27

**Authors:** Meghdad Eslami, Saba Khazeni, Xaniar Mohammadi Khanaghah, Mohammad Hossein Asadi, Mohamad Amin Ansari, Javad Hayati Garjan, Mohammad Hassan Lotfalizadeh, Mobina Bayat, Mohammad Taghizadieh, Seyed Pouya Taghavi, Michael R Hamblin, Javid Sadri Nahand

**Affiliations:** 1 Department of oral and maxillofacial surgery, Faculty of Dentistry, Tabriz University of Medical Sciences, Tabriz, Iran; 2 North Khorasan University of Medical Sciences, Bojnurd, Iran; 3 Department of Plant, Cell and Molecular Biology, Faculty of Natural Sciences, University of Tabriz, Tabriz, Iran; 4 Infectious and Tropical Diseases Research Center, Tabriz University of Medical Sciences, Tabriz, Iran; 5 Department of Pathology, School of Medicine, Tabriz University of Medical Sciences, Tabriz, Iran; 6 Student Research Committee, Kashan University of Medical Sciences, Kashan, Iran; 7 Department of Virology, Faculty of Medicine, Iran University of Medical Sciences, Tehran, Iran; 8 Laser Research Centre, Faculty of Health Science, University of Johannesburg, Doornfontein, 2028 South Africa

**Keywords:** Metastasis, MicroRNA, Long non-coding RNA, Circular RNA, Non-coding RNAs

## Abstract

Across the world, oral cancer is a prevalent tumor. Over the years, both its mortality and incidence have grown. Oral cancer metastasis is a complex process involving cell invasion, migration, proliferation, and egress from cancer tissue either by lymphatic vessels or blood vessels. MicroRNAs (miRNAs) are essential short non-coding RNAs, which can act either as tumor suppressors or as oncogenes to control cancer development. Cancer metastasis is a multi-step process, in which miRNAs can inhibit or stimulate metastasis at all stages, including epithelial-mesenchymal transition, migration, invasion, and colonization, by targeting critical genes in these pathways. On the other hand, long non-coding RNAs (lncRNAs) and circular RNAs (circRNAs), two different types of non-coding RNAs, can regulate cancer metastasis by affecting gene expression through cross-talk with miRNAs. We reviewed the scientific literature (Google Scholar, Scopus, and PubMed) for the period 2000–2023 to find reports concerning miRNAs and lncRNA/circRNA-miRNA-mRNA networks, which control the spread of oral cancer cells by affecting invasion, migration, and metastasis. According to these reports, miRNAs are involved in the regulation of metastasis pathways either by directly or indirectly targeting genes associated with metastasis. Moreover, circRNAs and lncRNAs can induce or suppress oral cancer metastasis by acting as competing endogenous RNAs to inhibit the effect of miRNA suppression on specific mRNAs. Overall, non-coding RNAs (especially miRNAs) could help to create innovative therapeutic methods for the control of oral cancer metastases.

## Introduction

Oral cancer is the 11th most frequent carcinoma worldwide, which has drawn interest from all across the world [1]. Oral cancer led to 177,757 new fatalities and 377,713 new cases in 2020 [2]. The majority of individuals are diagnosed with oral cancer at an advanced stage [3]. This late diagnosis leads to a poor prognosis and a greater prevalence of lymphatic metastasis [4, 5].

Differentiated oral squamous cell carcinoma (OSCC), which tends to spread to lymph nodes [6], is the 6th most frequent type of cancer in the world, with more than 200,000 new cases each year. OSCC occurs in three main anatomical sites: lip SCC (LSCC), tongue SCC (TSCC), and buccal mucosal SCC (BMSCC) [7, 8]. Males have morbidity and fatality rates of 6.6 and 3.1 per 100,000 persons, respectively, while females have rates of 2.9 and 1.4 per 100,000 persons. Also, the prevalence of OSCC is increasing in young caucasians between the ages of 18 and 44, especially in women [9, 10]. OSCC is a major problem for both individual health and socioeconomic well-being on account of risk factor exposure, limited treatment options, and high mortality.

Oral cancer carcinogenesis is influenced by a number of risk factors, like genetic variations, smoking, betel nut chewing, radiation exposure, and other lifestyle factors [11]. One additional potential risk factor for OSCC is infection with the human papillomavirus (HPV). As early as 2007, the International Agency for Research on Cancer recognized HPV16 as a risk factor for OSCC. OSCC has been linked to other sub-types of the virus such as HPV33, HPV35, that are also seen in cervical cancer [12]. Treatment for oral cancer may involve surgery, targeted therapy or chemotherapy depending on the tumor stage. Despite advances in treatment and detection methods over the last ten years, the five year survival rate, which averages between 45 and 50%, has not significantly increased [13]. Generally speaking, oral cancer still has a poor prognosis and a low survival rates [14]. It has a high likelihood of migration to nearby lymph nodes, adjacent tissues, and distant metastasis, and an unusually high chance of recurrence over the patient’s lifespan in those diagnosed with advanced-stage tumors [15].

MicroRNAs (miRNAs) are short (19–25 nt) single-stranded non-coding RNAs (ncRNAs) which specifically bind to the 3′ untranslated region (UTR) of the targeted gene mRNA to control its expression [16]. In light of the fact that numerous genes can be targeted by a single miRNA and a single target gene can have several miRNA binding sites, more than 60% of the genes in humans are thought to be controlled by miRNAs [17, 18]. Therefore, miRNAs play a critical role as regulators in almost all biology, including normal physiological and pathological processes, most notably cancer. It is accepted that miRNAs are crucial for preventing the growth and spread of cancer [19–21]. Additionally, increasing evidence suggests that the circRNA/lncRNA-miRNA-mRNA regulatory axis controls how oral cancer spreads [22–26]. In this overview, we summarize the role of specific miRNAs in the metastatic spread of oral cancer. Additionally, we discuss the function of lncRNA/circRNA-miRNA-mRNA networks in regulating signaling pathways and associated genes that are linked to the spread of oral cancer. These networks are becoming recognized as crucial regulators of carcinogenesis.

## Cancer metastasis

The process through which cancer cells detach themselves from the primary tumor, and go on to establish additional tumors at sites distant from the original tumor, is known as cancer metastasis. The initial tumor is generally not the principal reason for cancer death, but most fatalities are caused by metastasis. Around 9/10 of cancer deaths are caused by cancer metastasis, which also produces most morbidity [27, 28]. In 1889 Stephen Paget, a surgeon from the UK, first proposed the theory that the secondary sites of tumor dispersal are not due to chance alone, but instead, by an interaction between “seeds” (cancer cells) and the “congenial soil” (organs that certain tumor types spread to). This theory has now been widely accepted [29]. Up until recently, most cancer research was devoted to early tumor detection techniques and new therapeutic agents, as well as tumor growth inhibitors. If detected and treated early enough, the majority of solid tumors are now considered treatable or even curable, thanks to advances in early cancer detection and treatment. Howevert, once cancer has spread beyond the primary site, it is typically considered incurable and fatal [30, 31]. The mechanisms underlying the metastatic process are poorly understood, and in terms of preventing and controlling cancer metastasis, very little progress has yet been made.

There is still much to learn about the complex process of metastasis, which entails a number of sequential and connected steps as well as many biochemical reactions. The four crucial processes of detachment, invasion, migration, and adhesion all work togther in the formztion of metastases. Following their initial separation from the primary tumor, cancer cells initially migrate, invade, and travel through blood and lymphatic vessels to various locations. When the arrive at their destination, they settle (adhere), proliferate, and the secondary tumor spreads [32]. Multiple signaling mechanisms control metastatic growth, and the extracellular matrix (ECM) in the destination site has an efffect. The genes that respond to stress are now thought to act as metastatic genes to support inflammation, stress-induced wound healing, and angiogenesis [32, 33].

Prior to spreading, cells must initially detach from the primary tumor [28]. When normal cells endothelial or epithelial) become separated from their ECM, they undergo a type of cell death known as anoikis (cell death brought on by ECM detachment) [34]. Both the mitochondrial route of apoptosis and the death receptor pathways are engaged during anoikis [34]. Metastatic cancer cells are able create a defense against anoikis [35, 36]. The term “epithelial-mesenchymal transition” (EMT) describes the resistance of tumor cells to anoikis, as well as a number of other properties, including alterations in the adherence of cells and tissues, cell invasion and migration, and cell polarity. The majority of metastatic cells share the defining characteristics of EMT [36]. From being highly differentiated, polarized, and structured cells, epithelial cells are transformed into a mesenchymal-like immature state, as isolated cells with the potential for invasion and migration [37].

Two essential components of the metastatic cascade are the cell’s ability to migrate and invade. There are two different ways that metastatic cells can infiltrate through the ECM, mesenchymal (fibroblastoid) and amoeboid cellular migration. Protease-dependent enzyme activities are required for mesenchymal cell migration in order to break down the ECM structure and allow the cells to pass through. Mesenchymal cell migration can be stopped by blocking ECM-degrading proteases, e.g. matrix metalloproteinases (MMPs), which are important in wound healing. In the protease-independent method of amoeboid cell migration, cells use mechanical forces rather than enzymatic degradation to build a passageway. The ability to move chemotactically (to follow a chemokine gradient), total loss of cell polarity, and loose ECM attachment are characteristics of amoeboid cell invasion [36].

Single metastatic cancer cells or large clusters of cells can move throughout the body [36, 38]. In contrast to the collective migration of cell clusters, which only uses mesenchymal cell migration, both mesenchymal migration mediated by proteases and protease-independent amoeboid-like migration are capable of facilitating the movement of individual cancer cells. Epithelial cell polarity is lost and an EMT-induced mesenchymal state is produced in single cells, which leave a primary tumor from its periphery. A solid epithelial tumor may release one or more single cells as a result of EMT, which is characterized by interference with tight cell-cell junctions, adopting a fibroblastoid spindle-shaped morphology, increased cell-stromal interactions, and slower cell division rates and invasiveness [38].

Cells which migrate collectively maintain their cell-cell connections by ongoing production of adhesion molecules, in contrast to solitary cells that migrate on their own. They may migrate as unattached cell clusters or groups, and move as strands, tubes, sheets, or clusters (cohort migration), or they may keep their attachment to the parent tumor (coordinated invasion) [36]. Cancer cell migration in a clustered cohort appears to be very effective at obliterating lymphatic or blood vessels and preserving the cells under flow conditions. Mostly squamous cell carcinoma and basal cell carcinoma from various origins undergo collective cell migration [36, 38].

Throughout the past 30 years, major improvements have been made in understanding cancer metastasis at the molecular, cellular, and signaling pathway levels, thus opening up a variety of potential targets for preventing cancer metastasis. These may involve modifying the biochemical mechanisms and signaling pathways that control cell adhesion, dissociation, invasion, migration, and interaction with the tumor microenvironment [28]. The present review aims to provide a comprehensive explanation of how various miRNAs and other ncRNAs could regulate the process of invasion, migration, and adhesion in oral cancer cells.

## MicroRNA biogenesis

The stability and translation of messenger RNAs (mRNA) are controlled by a class of endogenous ncRNAs called microRNAs (miRNAs), which are 19–25 nucleotides (nt) in length. Ambros et al. originally discovered miRNAs in 1993, completely overturning previous theories about mRNA translation [39]. Canonical miRNA processing starts with RNA polymerase II converting a miRNA gene into a stem-loop-structured primary miRNA (pri-miRNA). The Drosha and DGCR8 microprocessor complex cleaves this pri-miRNA using endonuclease activity to produce a hairpin miRNA precursor with a 70 nt length [40, 41]. Exportin-5 facilitates the cytoplasmic entry of pre-export miRNAs [42], plus within the cytoplasm, the TRBP and Dicer(double-stranded) complex converts the pre-miRNA into a miRNA duplex. The complex that induces silencing via RNA appears to contain either both mature miRNA duplex strands, according to in vitro experiments (RISC). One strand of this duplex must be destroyed to cause the intended mRNA to be repressed, possibly depending on thermodynamic stabilities [43]. The mature miRNA is directed to a target mRNA by the RISC complex, and this complex contains the key component Argonaute 2, after strand selection. Here, the interactions promote target mRNA destabilisation and translational repression by cleaving the target mRNA or deadenylating (shortening the 3′ poly-A tail) the target mRNA [44, 45].

## Alterations in miRNA expression in cancer cells

George Calin, Carlo Croce, and associates reported the loss of miR-16 and miR-15a expression within B-cell chronic lymphocytic leukemia (CLL) in 2002, providing the first proof that human tumors express abnormal miRNAs [46]. There are no protein-coding genes in this area, which is lost in most B-cell CLL specimens. Since that first report miRNA expression in human cancers has been extensively studied to discover any new miRNAs associated with cancer. Many investigations have analyzed miRNA expression in various types of cancer, and reported miRNA profiless that differ in expression between healthy and malignant cells or tissues. In 334 samples of tumors and healthy tissue, Jun Lu, Todd Golub, et al. evaluated the expression of 217 miRNAs as well as 16,000 mRNAs. It was shown that a number of miRNAs were either up-regulated or down-regulated in cancer, and these miRNA expression patterns were more accurate in distinguishing different cancer types compared to mRNA expression patterns [47].

A variety of mechanisms, including chromosomal abnormalities, amplification, deletion, mutation, and translocation, can affect the production of miRNAs in human cancer. Activation or repression of transcription, epigenetic modifications, and structural flaws in the genes that produce miRNAs could also be involved. The Croce group examined the chromosomal position of various miRNAs, and found that miRNA genes are frequently located in those genetic regions that are altered in cancer [48]. These DNA sequences contain insertion sites or chromosomal translocation for tumor-associated viruses like HPV, regions of deletion that might encode tumor-suppressor genes, and regions of amplification that may contain oncogenes [49].

Chromosome 13q14 is where the miR-15a/miR-16-1 cluster is located, an area that is often eliminated in CLL. The family of miR-29 miRNAs, which is found in the areas 7q32 and 1q30 and is often lost in acute myeloid leukemia, is another example. Other factors in addition to genetic changes, are involved in the downregulation or deletion of particular miRNAs. For example, the mir-34 miRNA group was found to be epigenetically silenced via CpG island hypermethylation in a variety of cancer types, resulting in downregulation of miR-34 expression [49]. Unusual transcription factor activity may potentially contribute to deregulation of miRNA expression, which could involve either decreased or increased transcription from the miRNA gene. As one example, the tumor suppressor transcription factor p53, binds to the promoter for mir-34 and activates it. This may lead to miR-34 downregulation in those human cancers, where p53 is commonly altered or deleted. Moreover, p53 also favorably regulates the clusters miR-29 and miR-15a/miR-16-1 [50]. However, Oncoproteins which are frequently overexpressed or upregulated in human cancer, include certain transcription factors. For instance, the mir-29 group mir-26a and let-7a, every one of whom are downregulated in cancer, are transcriptionally repressed by MYC [49].

## MiRNAs as either tumor suppressors or oncomiRs in cancer

By affecting the expression of tumor oncogenes or tumor suppressors respectively, miRNAs can either promote or suppress the cancer phenotype. Typically, tumor-suppressor miRNAs are underexpressed in cancer, while tumors generally exhibit an overexpression of oncogenic miRNAs (oncomiRs). Depending on the type of cancer and the particular miRNA that is affected, cancer cell invasion, progression and/or survival could be drastically affected when these tumor-suppressor or oncomiR miRNAs are suppressed or activated, respectively. It is also conceivable that cancers can become totally dependent on, or “addicted” to, a specific oncomiR, in which case suppressing the oncomiR could cause the tumor to completely regress [51]. As a result, miRNAs can be categorized as either tumor-promoters or tumor-suppressors, and this distiction would govern whether to alter their expression for therapeutic purposes.

Nonetheless, there are arguments in favor of caution in therapeutic approaches involving miRNAs. There are contradictory reports in the literature about whether or not any particular miRNA is a tumor-suppressor or tumor-promoter. Some miRNAs have repeatedly been demonstrated to be tumor suppressive in one situation yet carcinogenic in another. Given the wide array of genes that any specific miRNA can affect, the variety of consequences is not surprising. It implies that any designation of a miRNA as tumor suppressive or oncogenic should always be carefully examined because it may constitute an oversimplification [52].

### MiRNAs as inhibitors of metastasis in oral cancer

Table 1 provides a summary of the main miRNAs which have been implicated in the process of metastasis. In 2007, it was first discovered that miRNAs and metastasis were connected to each other. Li Ma, Robert Weinberg, and coworkers evaluated the miRNA expression profiles in breast cancer cells, both metastatic and non-metastatic, as well as in healthy human mammary epithelial cells. This resulted in the discovery of numerous miRNAs linked to metastasis [53]. One of them, miR-101 also prevented the spread of oral cancer by inhibiting migration and invasion [54, 55].

The CX chemokine receptor 7 (CXCR7) recognizes its ligand CXCL12, and affects cell adhesion, viability, and tumor growth [56]. Furthermore, the stimulation of the CXCR7 signaling pathway caused by CXCL12 increases the proliferation, invasion and metastasis of tumor cells. CXCR7 is broadly expressed in many types of cancer [57, 58]. CXCL12 and CXCR7 are both concurrently increased in rapidly proliferating oral cancer [59]. Hui et al. [60] discovered that miR-101 could act as a tumor suppressor in OSCC, by targeting CXCR7. According to their findings, OSCC cell lines and tissues showed strongly increased expression of CXCR7 and downregulation of miR-101. Furthermore, OSCC tumor metastasis and growth were inhibited in vivo by either deletion of CXCR7 or overexpression of miR-101. Moreover, both in vitro and in vivo, miR-101 inhibited invasion and migration, which lowered OSCC metastasis [60]. It was later discovered that the expression of exosomal miR-101-3p, which was produced from human bone marrow mesenchymal stem cells (hBMSCs), could inhibit oral cancer invasion and migration by downregulating COL10A1 (type X alpha 1 chain of collagen) [61]. Different cellular processes and reactions are controlled by TGF-β signaling pathways, including proliferation, differentiation, cell death, and migration. TGF-β signaling operates in a different way depending on the tumor stage. In pre-malignant cells, it initially acts as a tumor suppressor, but as the disease progresses, it promotes invasion and metastasis [62]. Recent studies have also shown that miR-101 may play a significant role in TGF-R1 activity, as a miR-101-mediated modulator of OSCC cell motility by inhibiting TGF-R1-induced OSCC cell invasion and metastasis [63]. Additionally, the ectopic expression of miR-101 might block the AKT/mTOR pathway to attenuate the EMT in OSCC, providing a theoretical foundation for targeted treatment of OSCC to lower metastasis and recurrence and improve patient cure rates [64].

**Fig. 1:**
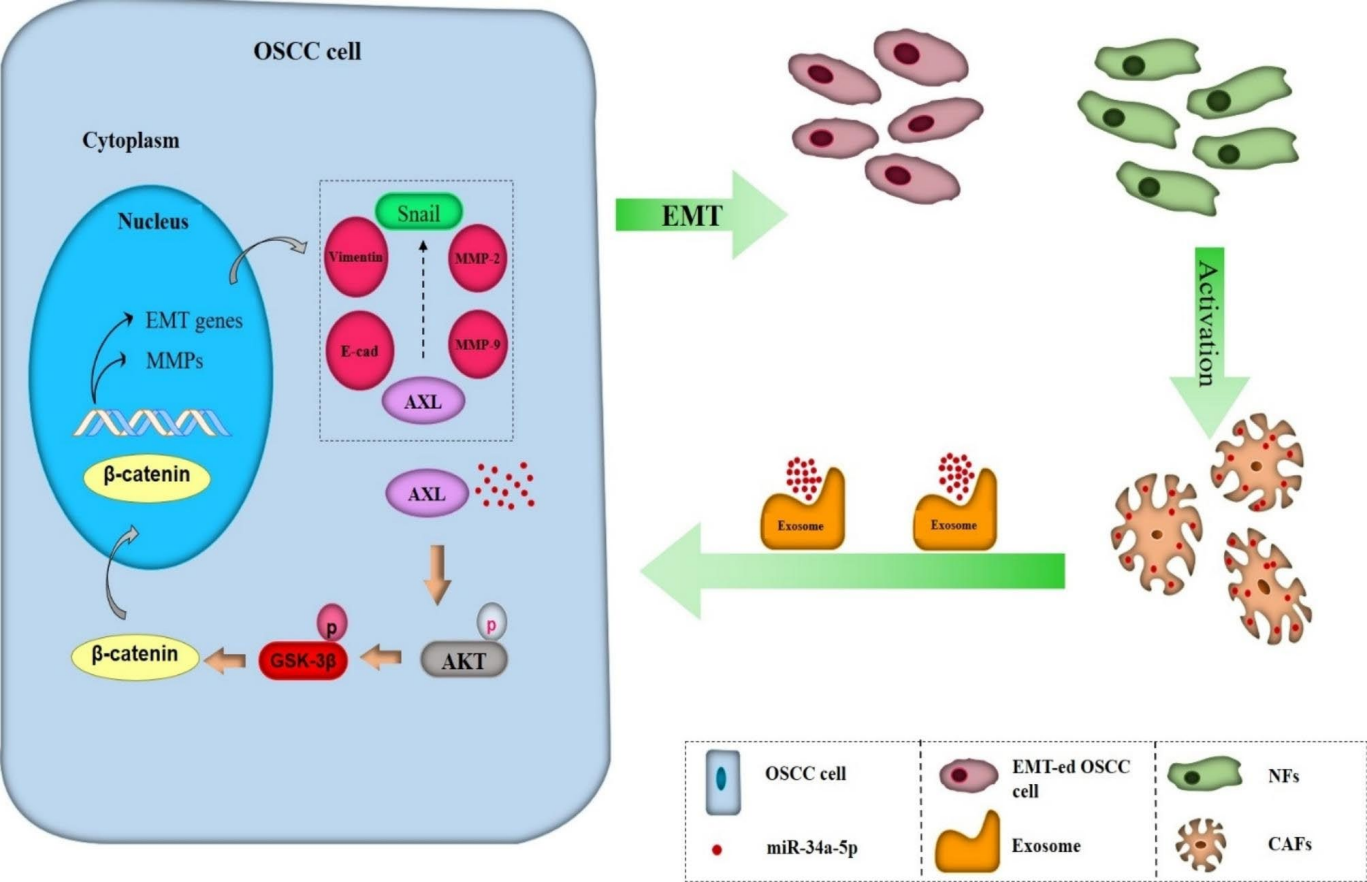
Exosomal miR-34a-5p can suppress metastasis of OSCC cells by targeting AXL. Transfer of CAF-derived exosomal miR-34a-5p to OSCC cells promotes by fibroblasts and miR-34a-5p inhibits metastasis and proliferation of OSCC cells through inhibiting EMT and MMP-2/9 activation by targeting AXL [65]

MiR-34a is the 2nd most investigated metastasis-associated miRNA, with a variety of functional roles that are related to metastasis (Table 1). According to reports, OSCC cells showed a dramatic downregulation of miR-34a expression [65–67]. On the other hand, increasing miR-34a expression could prevent OSCC cells from metastasizing by affecting MMP9 and MMP14 activity [66], as well as IL6R expression [67]. Interestingly, Li et al. [65] reported that oral cancer cells showed increased proliferation and metastasis as a result of miR-34a-5p exosome-mediated paracrine signaling in cancer-associated fibroblasts (CAFs). They discovered that exosomes produced from CAFs had much lower miR-34a-5p levels, and that fibroblasts could deliver exosomal miR-34a-5p into OSCC cells. Their findings showed that miR-34a-5p overexpression in CAFs could prevent OSCC cells from developing into tumors during mouse xenograft experiments. One direct downstream target of miR-34a-5p is AXL, to which it can bind in order to inhibit OSCC cell proliferation and metastasis. The miR-34a-5p/AXL axis could support the progression of OSCC via the AKT/GSK-3/β-catenin signaling pathway, and may promote the EMT to encourage the spread of oral cancer cells. Nuclear translocation of β-catenin was promoted by the miR-34a-5p/AXL axis, which also increased the Snail transcription factor. thus triggering MMP-9 and MMP-2. In oral cancer cells, the miR-34a-5p/AXL axis increased aggressiveness and could be a therapeutic target for OSCC by affecting AKT, GSK-3, β-catenin, and Snail signaling (Fig. 1) [65].

Recently, Dharavath et al. [68] reported that miR-944 showed potential anti-metastatic activity in vivo and in vitro. They observed that miR-944 inhibited migration, invasion, and EMT of TSCC cells by targeting MMP-10. Hence they suggested that this miRNA could be a therapeutic target in TSCC patients [68].

Cancer stem cells (CSCs), or tumor-initiating cells comprise a very small fraction of all the cells in a tumor. CSCs possess the ability of adult stem cells for self-renewal and differentiation. According to some theories, CSCs are mainly responsible for tumor growth, the initiation of invasion and metastasis, as well as recurrence [69, 70]. The development of both primary and metastatic cancers is caused by CSCs [71]. Tumorigenesis and metastasis have been reported to be inhibited by miRNAs which suppress CSC properties. In oral cancer, miR-495 could suppress tumor progression [72]. The regulation of miR-495 is disrupted in a variety of stem cells and cancer cells [73–75]. For example, in OSCC, miR-495 expression was markedly downregulated, and when it was expressed ectopically in CSCs, it reversed the EMT process, inhibited cellular migration, proliferation, and invasion, and promoted cell death via the HOXC6-mediated TGF-β signaling pathway [72].

**Fig. 2:**
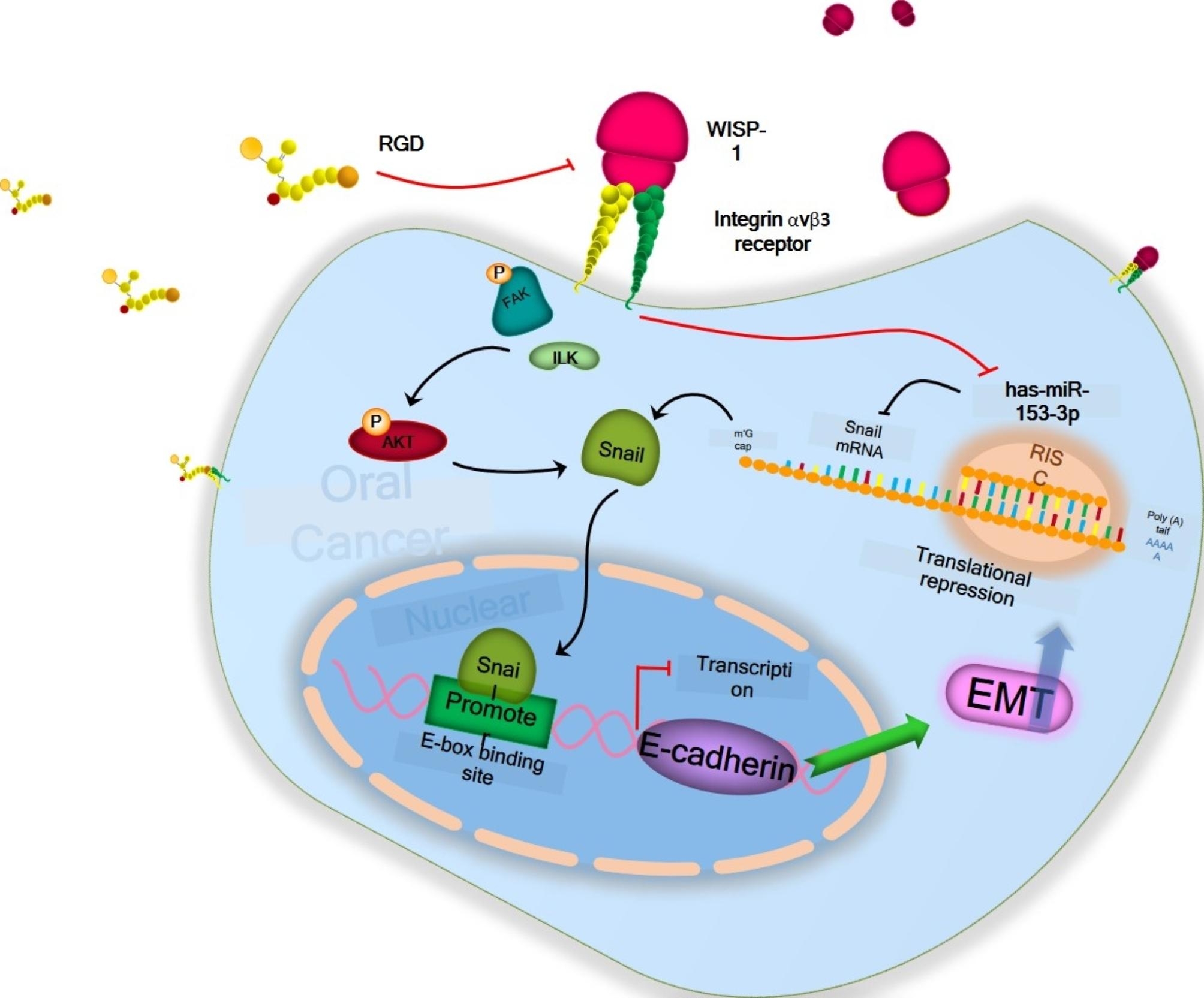
Regulation of EMT activation by miR-153-3p and WISP-1. WNT1-inducible-signaling pathway protein 1 (WISP-1) promotes the activation of EMT by upregulating Snail expression and regulating the integrin αvβ3/FAK/ILK/Akt signaling pathway. MiR-153-3p inhibits OSCC cell EMT and metastasis by targeting Snail, in return WISP-1 and induces metastasis [80]

As was previously mentioned, the mesenchymal-epithelial transition (MET), which is the opposite of the EMT process, encourages metastatic colonization in certain cancer types while facilitating tumor cell invasion and dissemination in carcinoma cells [76, 77]. According to several experimental studies, cancer cells interact with the chemokine (C-C motif) ligand 21 (CCL21)/chemokine (C-C motif) receptor type 7 (CCR7). This interaction (along with CXCL5/CXCR2) activates Snail and glycogen synthase kinase (GSk)-3 to promote the EMT [78]. Furthermore, it has been suggested that TGF-β interacts with the NF-kB signaling system to promote EMT in cancer cells [79]. In oral cancer, certain metastatic miRNAs control the EMT process. MiR-153-3p targets Snail in OSCC cells to block activation of EMT (Fig. 2) [80], while miR-532-3p targets CCR7 to reduce EMT, migration, and invasion [81]. Nevertheless, miR-940 overexpression prevented TSCC cells from metastasizing by inhibiting EMT, invasion and migration because it targeted the IL-8/CXCR2/NF-B pathway [82][83].

The process of angiogenesis governs tumor development and metastasis, and significantly promotes cancer growth [84]. Neuropilin 1 (NRP-1) is a vascular endothelial growth factor (VEGF) co-receptor [85, 86] that shows increased expression in OSCC [87, 88], and esophageal carcinoma [89]. NRP1 overexpression has been found to affect immunity, angiogenesis, tumor invasion, and growth [90, 91]. The molecular pathways of NRP1 activity, however, have not yet been clarified. Recently, multiple studies showed that NRP1 expression in human cancer may be controlled by a variety of miRNAs [87, 90, 92]. MiR1247 was found to target NRP1 and reduce cell growth in pancreatic cancer models both in vivo and in vitro [90]. Cui et al. [93] found that by targeting NRP1, miR-9 and miR-181b prevented vascular endothelial cells in oral cancer from migrating, adhering to one another, and forming tubes. This report was consistent with Shang et al. [94] who reported that ectopic expression of miR-9 led to the induction of apoptosis, cell cycle arrest, and inhibited invasion of OSCC cells by targeting CDK 4/6 pathways [94]. Moreover, Wu et al. demonstrated that miR-320 also carried out the same function [87]. Liu and colleagues [88] discovered that miR-338 targeted NRP1 in OSCC cells, and that overexpression of NRP1 could significantly reduce the ability of miR-338 to prevent proliferation in these cells. Moreover, miR-338 overexpression prevented OSCC metastasis by inhibiting NRP1, suggesting that miR-338 could be a possible therapeutic target in OSCC [88].

Recent research suggests that miRNAs may also be involved in lymphangiogenesis, the creation of new mature lymphatic tubes from already-existing lymphatic vessels. MiR-126 is the best example of a lymphangiogenic miRNA. MiR-126 downregulation was shown to support oral cancer angiogenesis and lymphangiogenesis by increasing the expression of the genes for VEGF-A and VEGF-C, respectively [95]. Another example is miR-300 which can regulate the interaction between WISP-1 (protein 1 of the WNT1-inducible signaling pathway) and CCN4 (a member of the CCN family of cysteine-rich matrix proteins) [96]. It was reported that WISP-1 is responsible for downregulating miR-300, resulting in increased VEGF-C production [96]. As a result, OSCC lymphatic vessel formation will be increased, which would encourage cancer metastasis. In contrast, WISP-1 also controls VEGF-A [96]. Further investigation into the relationship between WISP-1 and miR-126 would be interesting [97].

Recently, niclosamide (an anthelmintic medication) has been shown to decrease vasculogenic mimicry (VM) by affecting miR-124 expression levels [98]. VM is the ability of an aggressive tumor to spontaneously produce blood vessels containing tumor cells but without any endothelial cells. This will guarantee that there is a sufficient blood supply for the growth and metastasis of oral cancer [99]. Niclosamide could inhibit VM by upregulating miR-124, which in turn has anti-tumor effects [98].

Mammalian species have five Notch ligands (Jagged 1 & 2, and Delta-like 4, 3, & 1), as well as four Notch receptors (Notch 1, 2, 3 & 4). When Notch ligands and receptors interact, the signaling pathway is triggered. Two important proteolytic enzymes are involved in this process. The first is an enzyme that converts tumor necrosis factor (also known as ADAM, a distintegrin and metalloprotease), which breaks down the receptor extracellular region. Then secretase catalyzes the second hydrolysis process to release the Notch intracellular domain (NICD) in the nucleus, which then attaches to the transcription factor CSL to activate downstream genes [100]. It was discovered that OSCC cells and tissues overexpress Notch1, and it has been found that this overexpression was related to the clinical stage, TNM-stage, differentiation, degree of invasion, metastasis to lymph nodes, and localized recurrence [101–103]. According to studies, Notch1 activation increases OSCC cell migration, invasion, and EMT, while inhibiting apoptosis, [101, 104]. These findings suggest that treatment of OSCC by specifically targeting Notch1 could be beneficial. Recently, Lv et al. [74] showed that in vitro invasion and proliferation of OSCC cells were inhibited by exogenous expression of miR-495. They investigated the inhibitory mechanism of miR-495 activity. They found that Notch1 was a direct functional target of miR495 in OSCC. By reactivating Notch1, the inhibitory effects of miR495 on OSCC cell invasion and proliferation were reversed. Their findings revealed that miR495 played a major role in the control of OSCC metastasis, in part by targeting Notch1 [74].

In order to spread to distant regions of the human body, epithelial tumor cells lose their characteristic markers, such as cytokeratins and E-cadherin, and instead express proteins found in mesenchymal cells, such as vimentin, N-cadherin and fibronectin in a process called the EMT [105]. After spreading to distant regions of the body, these mesenchymal cancer cells can then undergo the MET (reverse of EMT), which enables them to revert to expressing epithelial markers. The “c-MET tyrosine kinase” is encoded by the c-MET proto-oncogene, and promotes tumor migration and metastasis, [106, 107], This could be a significant factor in treatment resistance in oral cancer [108–111]. c-MET is often increased in a range of cancers to encourage the spread and growth of tumors. A poor prognosis and increased tumor metastasis have been associated with expression of the HGF receptor, also known as c-Met [112, 113]. It has been suggested that CD44v3 might encourage phosphorylation of c-Met in response to HGF. Additionally, OSCC has an upregulation of CD44 v3 and c-Met [114–117]. The ability of OSCC cell lines to invade and migrate is closely linked to the overexpression of miR-143 [118]. Xu et al. [119] reported that miR-143 overexpression in OSCC cell lines may inhibit invasion and migration while having little to no effect on proliferation. OSCC often exhibits a significant downregulation of miR-143 expression. They also found that inhibition of CD44v3 decreased migration in OSCC cells, which was directly linked to the activation of c-Met via the CD44 v3/HGF signaling pathway. Additionally, miR-143 may target CD44v3 to prevent phosphorylation of c-Met, which would inhibit OSCC cell invasion and migration. This investigation suggested that miR-143 might play a role in a new treatment approach for OSCC.

**Table 1:** MiRNAs inhibiting the metastasis of oral cancer

Type of oral cancer	miRNA (expression)	miRNA Target	Inhibition/ Induction of metastasis	Samples	Results	Ref
OSCC	miR-23a-3p (Down)	Runx2	Inhibition	Human (OSCC tissues), In vitro (TSCCA & CAL-27)	Upregulation of miR-23a-3p inhibits metastasis of OSCC by targeting Runx2 and inhibiting PI3K/Akt signaling pathway	[120]
OSCC	miR-18a (Down)	HIF-1α	Inhibition	Human (OSCC tissues), In vitro (HSC-2 and YD-10B cell)	MiR-18a inhibits OSCC metastasis by targeting HIF-1α.	[121]
TSCC	miR-320b (Down)	IGF2BP3	Inhibition	Human (TSCC tissues), In vitro (SCC9 & CAL-27)	MiR-320b downregulation is related to lymphatic metastasis in TSCC patients. Upregulated miR-320b inhibits TSCC cell growth.	[122]
OSCC	miR-186 & miR-655 (Down)	PTTG1	Inhibition	In vitro (HSC2 & Ca9-22 cell)	MiR-186 and miR-655 regulate invasion by targeting PTTG1 & negatively regulating MMP-2 & MMP-9 activity.	[123]
OSCC	miR-655 (Down)	Metadherin	Inhibition	Human (TSCC tissues), In vitro (SCC9 & CAL-27)	MiR-655 may suppress metastasis by inhibiting invasion and PTEN/AKT pathway.	[124]
OSCC	miR‑198 (Down)	CDK4	Inhibition	Human (OSCC tissues), In vitro (SCC-9 & Cal-27 cell)	Overexpression of miR‑198 inhibits invasion, EMT and metastasis of OSCC by targeting CDK4.	[125]
OSCC	miR-489-3p	TWSIT	Inhibition	In vitro (SCC-4)	Ectopic miR-489-3p expression prevents OSCC invasion, migration, and metastasis by inhibiting TWSIT expression.	[126]
OSCC	miR-5580-3p (Down)	LAMC2	Inhibition	Human (OSCC tissues), In vitro (SCC-4 & Cal-27 cell)	MiR‑5580‑3p suppresses migration of OSCC by targeting LAMC2.	[127]
OSCC	miR-29c	SERPINH1	Inhibition	In vitro (SCC25 cells)	MiR-29c prevents migration, invasion, and metastasis of OSCC cells by targeting SERPINH1.	[128]
OSCC	miR-132	TGF-β1	Inhibition	Human (OSCC tissues), In vitro (CAL-27/CDDP)	MiR-132 prevents OSCC invasion and increases chemosensitivity to cisplatin by inhibiting TGF-β1.	[129]
OSCC	miR-504 (Down)	CDK-6	Inhibition	Human (OSCC tissues), In vitro (Cal27 & Tca8113 cells)	Upregulated miR-504 prevents OSCC migration and invasion by targeting CDK-6	[130]
OSCC	miR-519d (Down)	MMP3	Inhibition	Human (OSCC tissues), In vitro (HN30 & HN4 cells)	MiR-519d inhibits metastasis of OSCC cells by suppressing invasion and migration through targeting MMP-3.	[131]
OSCC	miR-149-5p (Down)	TGFβ2	Inhibition	Human (OSCC tissues), In vitro (CAL-27 & CAL-27/CDDP)	MiR-149-5p prevents OSCC cells from metastasizing by targeting TGFβ2.	[132]
TSCC	miR-149 (Down)	Specificity protein 1	Inhibition	Human (TSCC tissues), In vitro (CAL-27 & Tca8113 cells)	MiR-149 can prevent metastasis of TSCC cells by inhibiting invasion and migration by targeting specificity protein 1	[133]
OSCC	miR-365-3p (Down)	EHF	Inhibition	Human (OSCC tissues), In vitro (OC-3-IV-M), In vivo (CB17-SCID mice)	MiR-365-3p prevents OSCC cells from metastasizing by targeting EHF.	[134]
OSCC	miR-107 (Down)	TRIAP1	Inhibition	In vitro (OSC-4 and CAL-27 cells)	Upregulated miR-107 may suppress metastasis of OSCC cells by inhibiting migration through targeting TRIAP1.	[135]
OSCC	miR-532-3p (Down)	CCR7	Inhibition	Human (OSCC tissues), In vitro (CAL-27 & TCA8113 cells)	MiR-532-3p decreases invasion, migration, and OSCC EMT by targeting CCR7.	[81]
TSCC	miR-940	CXCR2	Inhibition	In vitro (TSCCA & Tca8113 cells)	Overexpressed miR-940 may inhibit metastasis of TSCC cells by inhibiting invasion, migration and EMT process through targeting IL-8/CXCR2/NF-κB axis.	[82]
TSCC	miR-320a (Down)	MRP2	Inhibition	Human (TSCC tissues), In vitro (SCC4 cells)	MiR-320a can suppress metastasis of OSCC cells by inhibiting migration and invasion by targeting MRP2.	[136]
OSCC	miR-300 (Down)	-	Inhibition	Human (OSCC tissues), In vitro (Cal-27 & Tca8113 cells)	MiR-300 inhibits metastasis of OSCC cells by suppressing EMT.	[137]
OSCC	miR-34a-5p (Down)	AXL	Inhibition	In vitro (SCC1 & CAL27 cells), In vivo (BALB/c nude mice)	MiR-34a-5p can prevent OSCC cells from metastasizing by targeting AXL.	[65]
TSCC	miR-34a	MMP9 & MMP14	Inhibition	Human (TSCC tissues), In vitro (CAL27 cells)	MiR-34a suppresses metastasis of TSCC cells by inhibiting invasion and migration through targeting MMP9 and MMP14.	[66]
OSCC	miR-34a (Down)	IL6R	Inhibition	Human (OSCC tissues), In vitro (Tca8113 cells)	Ectopic expression of miR-34a prevents metastasis of OSCC cells.	[67]
OSCC	miR-1-3p (Down)	DKK1	Inhibition	Human (OSCC tissues), In vitro (SCC-4 cells)	MiR-1-3p suppresses metastasis of OSCC cells by inhibiting migration through DKK1.	[138]
OSCC	miR-218-5p	CD44	Inhibition	In vitro (UM-SCC6)	MiR-218-5p may inhibit metastasis of OSCC cells by suppressing invasion through targeting CD44-ROCK pathway.	[139]
OSCC	miR-375 (Down)	PDGF‑A	Inhibition	In vitro (UM1 & CAL‑27)	MiR-375 targets PDGF-A to prevent OSCC cells from metastasizing by reducing invasion and migration.	[140]
OSCC	miR-376c-3p (Down)	HOXB7	Inhibition	Human (OSCC tissues), In vitro (SCC-25 cells)	MiR-376c-3p inhibits metastasis of OSCC cells by reducing invasion and migration through targeting HOXB7.	[141]
OSCC	miR-376c	-	Inhibition	Human (OSCC tissues), In vitro, In vivo	Upregulated miR-376c inhibits invasion, migration and metastasis of OSCC cells via regulating RUNX2-INHBA axis.	[142]
OSCC	miR-377 (Down)	HDAC9	Inhibition	Human (OSCC tissues), In vitro (UPCI-SCC-116cells)	MiR-377 can inhibit metastasis by suppressing migration through targeting HDAC9.	[143]
OSCC	miR-143 (Down)	HK2	Inhibition	Human (OSCC tissues), In vitro (Tca8113 cells & OECM-1)	MiR-143 can suppress metastasis of OSCC cells by inhibiting invasion and migration through targeting HK2.	[118]
OSCC	miR-143 Down)	CD44 v3	Inhibition	Human (OSCC tissues), In vitro (Tca-8113 & CAL-27 cells)	Upregulated miR-143 inhibits invasion and migration in Tca-8113 and CAL-27 cells.	[119]
OSCC	miR-195-5p (Down)	TRIM14	Inhibition	Human (OSCC tissues), In vitro (Cal27 & Tca83 cells)	MiR-195-5p prevents OSCC cells from migrating and invading by targeting TRIM14.	[144]
Oral cancer	miR-30 family	mGluR5	Inhibition	In vitro (SDF-1 stimulated B88 cells)	MiR-30 family may inhibit metastasis of oral cancer by targeting mGluR5.	[145]
OSCC	miR-101 (Down)	CXCR7	Inhibition	Human (OSCC tissues), In vitro (SCC-9, Fadu, Cal-27, & SCC-4 cells)	OSCC metastasis can be prevented by overexpression of miR-101 because it blocks migration and invasion via CXCR7.	[146]
OSCC	miR-20a (Up)	-	Inhibition	Human (OSCC tissues), In vitro (Cal27 cells)	MiR-20a is elevated in HPV positive OSCCC samples. MiR-20a is overexpressed by HPV-16 E7, which prevents Cal27 cells from migrating and invading.	[147]
OSCC	miR-204-5p (Down)	CXCR4	Inhibition	Human (OSCC tissues), In vitro (Cal27 cells)	MiR-204-5p suppresses metastasis in OSCC cells.	[148]
OSCC	miR-204 (Down)	SLUG and SOX4	Inhibition	In vitro (SAS & OECM1 cells), In vivo (nude mice)	MiR-204 is decreased in tissues from lymph node metastases. MiR204 disrupts EMT properties of OSCC cells.	[149]
OSCC	miR-23b & miR-27b (Down)	-	Inhibition	Human (OSCC tissues), In vitro (SAS & HSC3 cells)	Invasion and migration of OSCC cells are inhibited by upregulation of miR-23b or miR-27b.	[150]
OSCC	miR-216a (Down)	EIF4B	Inhibition	Human (OSCC tissues), In vitro (SCC-4 & CAL 27 cells)	By targeting EIF4B, miR-216a prevents OSCC cells from metastasizing.	[151]
OSCC	miR-338 (Down)	NRP1	Inhibition	Human (OSCC tissues), In vitro (SCC-15 & Tca-8113 cells)	MiR-338 prevents OSCC cells from metastasizing by inhibiting NRP1.	[88]
OSCC	miR-433 (Down)	HDAC6	Inhibition	Human (OSCC tissues), In vitro (SAS & HSC2 cells), In vivo (BALB/c nude mice)	MiR-433 targets HDAC6. When miR-433 is expressed ectopically it prevents OSCC cells from migrating and invading.	[152]
OSCC	miR-506	GATA6	Inhibition	Human (OSCC tissues), In vitro (SCC-4 & SCC-9 cells)	MiR-433 prevents OSCC cells from migrating and invading by targeting GATA6.	[153]
OSCC	miR-320 (Down)	NRP1	Inhibition	Human (OSCC tissues), In vitro (OC2 & HUVECs), In vivo (mice)	MiR-320 targets NRP1 to prevent OSCC cells from migrating.	[87]
OSCC	miR-101 (Down)	AKT	Inhibition	Human (OSCC tissues), In vitro	Upregulated miR-101 decreases EMT and colony formation by suppressing AKT/mTOR.	[64]
OSCC	miR-101 (Down)	TGF-βR1	Inhibition	Human (OSCC tissues), In vitro (Tca8113 & SCC-9 cells)	OSCC metastasis is reduced by miR-101 by inhibiting TGF-βR1.	[154]
OSCC	miR-101-3p (Down)	COL10A1	Inhibition	Human (OSCC tissues), In vitro (TCA8113 cells)	Upregulated miR-101-3p may reduce OSCC metastasis by inhibiting migration and invasion.	[61]
OSCC	miR-142 miR-128 (Down)	HOXA10	Inhibition	Human (OSCC tissues), In vitro (SSC-25 & SCC-9 cells)	Upregulation of miR-128 and miR-142 suppressed metastasis of OSCC by targeting HOXA10.	[155]
OSCC	miR-153-3p	Snail	Inhibition	Human (OSCC serum), In vitro (SCC4 cells)	MiR-153-3p suppressed activation of EMT via targeting Snail.	[88]
OSCC	miR-495 (Down)	Notch1	Inhibition	Human (OSCC tissues), In vitro (SCC-9, CAL-27, & Tca8113)	MiR-495 may inhibit metastasis by suppressing OSCC cell invasion by targeting Notch1.	[74]
OSCC	miR-137 (Down)	BRD4	Inhibition	Human (OSCC tissues), In vitro (Cal-27, SCC1 & SCC4)	Upregulated miR-137 promotes metastasis by increasing OSCC cell migration and invasion	[156]
OSCC	miR-9 (Down)	CDK 4/6	Inhibition	Human (OSCC tissues), In vitro (Tca8113)	Upregulated miR-9 induces apoptosis, cell cycle arrest and inhibits invasion of OSCC cells by targeting CDK 4/6 pathways.	[94]
OSCC	miR-144/451a cluster (Down)	-	Inhibition	In vitro (UM-SCC083A & UPCI-SCC029B cells)	The miR-144/451a cluster prevents OSCC cells from migrating, invading, and metastasizing by lowering MIF and CAB39 levels.	[157]
Oral cancer	miR-30a-5p (Down)	Fibroblast activation protein α (FAP)	Inhibition	Human (oral cancer patients), In vitro (SCC-15 & Tca-8113 cells)	MiR-30a-5p prevents OSCC cells from metastasizing by reducing invasion and migration through targeting FAP.	[158]
TSCC	miR-29b (Down)	Sp1	Inhibition	Human (TSCC tissues), In vitro (SCC-15 and CAL27)	MiR-29b reduces the invasion and migration of TSCC cells by targeting Sp1.	[159]
TSCC	miR-200a	DEK	Inhibition	In vitro (SCC15 cells)	Ectopic miR-200a expression prevents TSCC from migrating and invading by targeting DEK.	[160]
TSCC	miR-27b (Down)	ITGA5	Inhibition	In vitro (SCC-9 & CAL 27 cells)	When MiR-27b is upregulated, it inhibits TSCC cell migration by targeting ITGA5, which stops the EMT process.	[161]
TSCC	miR-200b or miR-15b (Down)	BMI1	Inhibition	In vitro (SCC25-res & CAL27-res cells), In vivo (TSCC xenograft mice)	Upregulated miR-200b or miR-15b suppresses metastasis in chemo-resistant TSCC xenografts by targeting BMI1.	[162]
TSCC	miR-944	MMP10	Inhibition	In vivo (mice), In vitro (AW13516 cell)	Upregulated miR-944 inhibits EMT, invasion, and migration of TSCC cells by targeting MMP10.	[68]

### MiRNAs as promoters of metastasis during oral cancer

While some miRNAs promote metastasis, the bulk of those that have been investigated so far have inhibitory effects (Table 2).

One of the most well known tumour suppressors in squamous cell carcinoma is called phosphatase and tensin homolog deleted on chromosome 10 (PTEN), a phosphatase enzyme whose primary substrate is phosphatidylinositol 3,4,5-trisphosphate (PIP3) [163, 164]. PTEN controls the PI3K/AKT pathway via phosphatidylinositol 3-kinase [165]. When AKT is phosphorylated it becomes activated, and then controls cell death, migration and invasion [166]. PIP3 is dephosphorylated by PTEN, which converts it into phosphatidylinositol-4,5-bisphosphate (PIP2), which inactivates AKT by dephosphorylating it. This regulation of PTEN can have anti-cancer effects. According to earlier research, PTEN expression is suppressed by some miRNAs, which also contribute to the growth of cancer [167, 168]. Lizumi and associates [169] demonstrated that miR-142-5p increased the amount of active AKT by targeting PTEN (p-AKT). This shows that PTEN effectively inhibits PIP3 dephosphorylation to accelerate cancer growth by regulating the PI3K/AKT pathway. Additionally, in OSCC cells, PTEN expression was decreased when miR-142-5p was overexpressed which then stimulated proliferation and invasion. Additionally, PTEN-deficient OSCC cells exhibited the same behavior as cells that had been treated with a miR-142-5p mimic [169]. Various miRNAs stimulate the PTEN/AKT pathway to inhibit the invasion and growth of many cancer types [170]. According to additional research, increased miR-655 expression prevented OSCC cells from proliferating and invading while also preventing the PTEN/AKT pathway from becoming activated [124, 171]. Zheng et al showed that the miR-24 seed sequence bound to the 3’ UTR of PTEN mRNA, thereby inhibiting PTEN translation by activating the PI3K/Akt pathway. The AKT pathway is involved in the control of PTEN expression by miR-24. MiR-24 increases cell invasion, viability, and chemoresistance via targeting the PTEN/Akt pathway [172]. Moreover, OSCC patients have higher miR-155 levels, and BMSCs displaying increased expression of miR-155 might promote the proliferation and spread of OSCC by inhibiting PTEN12 [173].

While certain miRNAs, such as miR-29b-1-5p, target E-cadherin directly to promote the EMT, other miRNAs indirectly stimulate the EMT [174]. For example, miR-134 targets PDCD7 thereby decreasing E-cadherin expression, promoting EMT, and stimulating oral cancer metastastasis [175]. Li et al. [176] asked whether or not miR-424 or miR-19a could regulate the expression of the TGFBR3 gene (transforming growth factor beta receptor 3). They discovered that in CAL-27 cells, miR-19a and miR-424 overexpression facilitated migration and EMT. When CAL-27 cells were transfected with a plasmid expressing the TGFBR3 gene, this reversed the increased migration that was induced by miR-19a or miR-424, and prevented EMT from progressing by decreasing p-p65 expression in comparison to the control group. These findings demonstrated that miR-424 or miR-19a overexpression could trigger EMT and encourage cell migration by targeting TGFBR3 [176].

Dickkopf-1 (DKK1) is a transcriptional target of the Wnt/β-catenin pathway, and acts as a suppressor of Wnt signaling [177]. According to research, lithium chloride usually encourages the spread of cancer cells, while Wnt signaling suppression via DKK1 reduces invasion in many cancers [177]. Since antagonist molecules may negatively control the Wnt/β-catenin signalling pathway, miRNAs that target these molecules could act as EMT drivers. It was discovered that the DKK gene complex (DKK1-4) inhibited tumor migration and invasion via negatively regulating β–catenin [178, 179]. It was found that DKK1 was targeted by miR-373-3p in TSCC tissues. MiR-373-3p increased TSCC metastases caused by EMT and constitutively activated Wnt/β-catenin signaling by specifically targeting DKK1 [180]. Secreted frizzled-related protein 1 (SFRP1) is a Wnt signaling antagonist, which interacts with Wnt proteins via its CRD domain, in contrast to the transmembrane frizzled receptor [181]. It was discovered that miR-27a-3p targeted SFRP1 and initiated EMT in OSCC stem cells [182].

The metastasis suppressor gene known as metastasis suppressor-1 (MTSS1), also referred to as MIM (missing in metastasis), was first shown to be a tumor suppressor gene in non-metastatic bladder cancer cell lines. Its expression is restricted to chromosome 8q24.1 in humans [183]. In metastatic cells, MTSS1 expression is typically diminished, whereas its relative expression is unclear in primary cancers. For example, it was confirmed that MTSS1 expression is decreased in esophageal, ovarian, prostate, colorectal, and breast cancers [184, 185], although hepatocellular carcinoma and breast cancer were found to have higher levels of MTSS1 expression [186, 187]. Recent studies revealed that both bladder and kidney cancer displayed low or nonexistent MTSS1 protein staining [188, 189]. The MTSS1 gene was found to be down-regulated in TSCC tissues. According to functional and mechanistic studies, the MTSS1 protein might be associated with the spread of cancer to various organ sites, most likely by interaction with the actin cytoskeleton, or by being regulated by miRNAs [190–192]. Guo et al. [193] analyzed the effect of the MTSS1 gene on the proliferation and invasion of Tca8113 cells using MTT, scratch wound healing, and invasion assays. They also examined whether miR-96 targeted MTSS1 and how it affected the biological changes caused by the MTSS1 gene in Tca8113 cells. They discovered that Tca8113 cells and TSCC tissues both showed down-regulated MTSS1 expression. Moreover, forced expression of MTSS1 resulted in reduced numbers of migrating cells, slower wound healing, and hindered proliferation. Moreover, Tca8113 cell proliferation and spread may be regulated by miR-96 through the activity of MTSS1. MiR-96 was unable to fully reverse Tca8113 cell propensity to invade. Therefore, they hypothesized that miR-96 targeting and MTSS1 suppression may hasten the development of TSCC by evading the regulation of proliferation and metastasis. Another study looked at connections between MTSS1 and miR-182-5p in OSCC [194]. Higher TNM grades were linked to elevated miR-182-5p expression in OSCC, so miR-182-5p was proposed to be involved in invasion and migration of OSCC. MiR-182-5p directly targeted MTSS1, and by down-regulating MTSS1 expression levels it may promote invasion and migration in oral cancer [194].

Cyclin-dependent kinases (CDKs). are sequentially activated and inactivated during the cell cycle progression. Cyclin-CDK inhibitors inactivate CDKs and work together with positive regulators (cyclins) to activate them. One cyclin-CDK inhibitor is P27 (also known as CDKN1B, KIP1, or cyclin-dependent kinase inhibitor 1B), and prevents the cell cycle from entering the S-phase. Ubiquitin-mediated protein degradation controls the levels of p27 at the post-translation level [195]. The substrate recognition element that binds to and marks p27 for ubiquitination and eventual degradation was identified as the F-box protein SKP2 [195]. In certain malignancies, low levels of p27 have been linked to faster tumor growth and a poor prognosis [195, 196]. MiRNA-221 was found to target CDKN1B (cyclin-dependent kinase inhibitor 1B, also known as p27) [197][198]. Moreover, CDKN1B/p27 has been suggested to be a potential therapeutic target and prognostic marker in ovarian cancer [199–201]. Yang et al. [202], investigated how miR-222-3p affected cell division, invasion, migration and apoptosis. They found that compared to healthy tissues, OSCC tissues had higher levels of miR-222-3p. Also, they claimed that miR222-3p might inhibit cell division, migration, and invasion as well as cause the death of Tca-83 and SCC-15 cells. Moreover, testing with luciferase reporters showed that miR-222-3p specifically targeted CDKN1B in OSCC cells. As a result, OSCC cell migration, invasion, and proliferation were reduced while the rate of cell death was increased when CDKN1B was overexpressed. Overall, they demonstrated that miR-222-3p, which targeted CDKN1B, caused OSCC cells to invade and metastasize by acting as an oncomiR, and could be used as a predictive biomarker in OSCC patients [202]. Chen et al. [203] found that OSCC tissues showed overexpression of miR-222, and that by targeting CDKN1, it could promote invasion and metastasis [203].

The Fbxw7 protein, also known as Sel-10, hCdc4, or hAgo, is a substrate for the ubiquitin ligase complex called Skp1-Cul1-F-box protein-Rbx1 (SCF), which binds to its receptor [204]. SCF is an E3-ubiquitin ligase which ubiquitinates certain proteins and leads to proteasome degradation [204, 205]. Moreover, FBXW7 controls many biological processes, such as cell cycle progression, differentiation, and stemness of brain cells, maintenance of genomic stability, and cell proliferation [206]. In a number of human cancers, FBXW7 acts as a tumor suppressor. Recent studies suggested that FBXW7 contributes to tumor metastasis, because increasing FBXW7 reduces cancer metastasis and EMT [204, 207]. FBXW7 was recently discovered to be a target gene for some miRNAs in various cancers. For example, miR-223 controlled acute lymphoblastic leukemia by reducing FBXW7 expression [208]. MiR-27a increased lung cancer cell growth by inhibiting FBXW7, demonstrating that FBXW7 could act as a tumor suppressor [209]. In TSCC cells, proliferation, migration, and invasion were markedly reduced when miR-24 was inhibited. When FBXW7 was suppressed, this caused TSCC cells to proliferate, migrate, and invade more readily. Conversely, when FBXW7 was restored, it significantly reduced the oncogenic effect of miR-24. They concluded that miR-24 could target FBXW7 especially in TSCC cells. As a result, miR-24 can promote TSCC cell metastasis by targeting FBXW7 and increasing migration and invasion [210]. In a different investigation, Jiang et al. demonstrated that by reducing the expression of FBXW7 in OSCC cells, miR-223 could encourage OSCC cell migration [211].

**Table 2:** MiRNAs promoting metastasis in oral cancer

Type of oral cancer	miRNA (Expression)	Target of miRNA	Inhibition/ Induction of metastasis	Samples	Results	Ref
TSCC	miR-675-5p (Up)	-	Induction	In vitro (SCC9 cell)	MiR-675-5p ectopic expression caused TSCC to metastasize.	[212]
TSCC	miR-17-5p	-	Induction	In vitro (CAL-27 cells)	MiR-17-5p may promote metastasis of TSCC cells via increasing migration.	[213]
OSCC	miR-222 (Up)	CDKN1B	Induction	Human (OSCC tissues), In vitro (TCA-83, CAL-27, SOC-9 & CAL-27)	MiR-222 may promote OSCC metastasis, and invasion by targeting CDKN1B.	[203]
OSCC	miR-155 (Up)	PTEN12	Induction	Human (OSCC tissues), In vitro	PTEN12 is a target of overexpressed miR-155, which causes OSCC cells to metastasize.	[173]
OSCC	miR-626 (Up)	RASSF4	Induction	Human (OSCC tissues), In vitro (HSC2 & Ca9-22 cell)	MiR-626 expression was significantly correlated with lymph node metastasis. Uppregulated miR-626 promotes EMT, migration, and invasion of OSCC cells by targeting RASSF4.	[214]
OSCC	miR-5100 (Up)	SCAI	Induction	In vitro (TCA-8113 cells)	MiR-5100 promotes invasion and migration of OSCC cells by targeting SCAI.	[215]
OSCC	miR-142-5p	PTEN	Induction	Human (OSCC tissues), In vitro (HSC3-M3 & SAS cells)	Upregulated miR-142-5p increased OSCC invasion by targeting PTEN.	[169]
OSCC	miR-211 (Up)	Bridging integrator-1 (BIN1)	Induction	Human (OSCC tissues), In vitro (HN6, HN4m SCC6, 9, & 25 cells)	MiR-211 may induce metastasis by promoting migration and invasion.	[216]
OSCC	miR-133	PDE1C	Induction	In vitro (SAS & OSC20)	MiR-133 promotes EMT phenotype.	[217]
OSCC	miR-182-5p (Up)	MTSS1	Induction	Human (OSCC tissues), In vitro (Tca8113 & Cal27 cells)	MiR-182-5p promotes invasion and migration of OSCC cells by targeting MTSS1.	[218]
OSCC	miR-222-3p (Up)	CDKN1B	Induction	Human (OSCC tissues), In vitro (SCC-15 & Tca-83 cells)	MiR-222-3p promotes invasion and migration of SCC-15 and Tca-83 cells by targeting CDKN1B.	[202]
OSCC	miR-146b (Up)	HBP1	Induction	Human (OSCC tissues), In vitro (SCC25 & SCC9 cells)	MiR-146b promotes SCC25 and SCC9 cell invasion and migration by targeting HBP1.	[219]
OSCC	miR-155-5p (Up)	ARID2	Induction	Human (OSCC tissues), In vitro (Cal27 & HN4)	Patients with OSCC had a positive correlation between miR-155-5p levels and lymph node metastases. MiR-155-5p promotes OSCC cell invasion and migration by targeting ARID2.	[220]
OSCC	miR-223-3p (Up)	SHOX2	Induction	Human (OSCC tissues), In vitro (Tca-8113, SCC-9, Cal-27, & SCC-25 cells)	MiR-223-3p causes OSCC cells to metastasize by targeting SHOX2.	[221]
OSCC	miR-223 (Up)	FBXW7	Induction	Human (OSCC tissues), In vitro (SCC15 & OECM1 cells)	MiR-223-3p may induce metastasis of OSCC cells by increasing migration by decreasing FBXW7 expression.	[211]
OSCC	miR-29b-1-5p (Up)	Cadherin-1	Induction	Human (OSCC tissues), In vitro (KON cell)	MiR-29b-1-5p could enhance metastasis of OSCC cells by inducing EMT.	[174]
OSCC	miR-29b	CX3CL1	Induction	In vitro (TW2.6 MS-10 cells), In vivo (SCID mice injected with SAS/amiR-29b#4 cells)	MiR-29b induces migration of OSCC cells by decreasing CX3CL1 expression.	[222]
OSCC	miR-134	PDCD7	Induction	Human (OSCC tissues), In vitro (SAS and OECM1 cells)	MiR-134 can induces metastasis by inhibiting migration and EMT by decreasing expression of PDCD7 and E-cadherin.	[175]
OSCC	miR-200c-3p	-	Induction	In vitro (SQUU-B & SQUU-A cells)	MiR-200c-3p may contribute to metastasis by promoting invasion in the OSCC microenvironment.	[223]
OSCC	miR-654-5p (Up)	Grb2-related adaptor protein (GRAP)	Induction	Human (OSCC tissues), In vitro (CAL-27/CDDP & Tca-8113/CDDP)	MiR-654-5p promotes metastasis of OSCC cells by increasing invasion and migration by regulating GRAP-mediated Ras/MAPK signaling.	[224]
OSCC	miR-625-3p (Up)	SCAI	Induction	Human (OSCC tissues), In vitro (WSU-HN6 & SCC25 cells)	MiR-625-3p may encourage migration by targeting SCAI to cause OSCC cells to metastasize.	[225]
OSCC	miR-196b (Up)	-	Induction	Human (OSCC tissues), In vitro (SAS & CAL-27 cells)	In SAS and CAL-27 cells, ectopic expression of miR-196b promotes EMT, migration, and invasion.	[226]
Oral cancer	miR-196a and b	-	Induction	Human (oral cancer tissues), In vitro (OECM1 & SAS cells)	MiR-196a and miR-196b induce migration and invasion of OECM1 and SAS cells by regulating the NME4-JNK-TIMP1-MMP signaling pathway.	[227]
OSCC	miR-448 (Up)	MPPED2	Induction	Human (OSCC tissues), In vitro (CAL-27 cells)	Silencing miR-448 leds to inhibition of migration in Cal-27 cells.	[228]
OSCC	miR-424-5p (Up)	SOCS2	Induction	Human (OSCC tissues), In vitro (OEC-M1 and SCC-9 cells)	MiR-424-5p promotes OSCC cell migration and invasion by targeting SOCS2.	[229]
OSCC	miR-187 (Up)	BARX2	Induction	In vitro (SAS cells)	When MiR-187 was expressed ectopically, it promoted OSCC cell migration.	[230]
OSCC	miR-155	-	Induction	Human (OSCC tissues), In vitro (CAL27 cells )	MiR-155 induces invasion and migration of OSCC cells by regulating BCL6/cyclin D2 axis.	[231]
OSCC	miR-221 (Up)	MBD2	Induction	In vitro (CAL-27 & UM1 cells),	MiR-221 expression was increased in metastatic cells compared to less metastatic OSCC cell lines. Downregulated miR-221 inhibits invasion and migration of metastatic cell lines (UM1 cells).	[232]
Oral cancer	miR-518c-5p (Up)	-	Induction	In vitro (B88-SDF-1 cells & CAL27 cells), In vivo (BALB/c nude mice)	Upregulated miR-518c-5p promotes migration and metastasis of OSCC cells.	[233]
TSCC	miR-21 (Up)	DKK2	Induction	Human (TSCC tissues), In vitro (SCC25)	MiR-21 induces invasion of oral cancer cells by targeting DKK2.	[234]
TSCC	miR-96	MTSS1	Induction	In vitro (Tca8113 cells),	MiR-96 promotes metastasis of Tca8113 cells by targeting MTSS1.	[193]
TSCC	miR-424 & miR-19a (Up)	TGFBR3	Induction	Human (TSCC tissues), In vitro (CAL-27 cells)	Upregulated miR-19a and − 424 induce EMT and migration of TSCC cells.	[83]
TSCC	miR-373-3p (Up)	DKK1	Induction	Human (TSCC tissues), In vitro (SCC-9 & UM1 cells)	MiR-373-3p induces metastasis and EMT by targeting the Wnt/β-catenin pathway.	[235]
TSCC	miR-24 (Up)	FBXW7	Induction	Human (TSCC tissues), In vitro (SCC15 & SCC25 cells)	MiR-24 can promote metastasis of TSCC cells by inducing migration and invasion by targeting FBXW7.	[210]
TSCC	miR-24	PTEN	Induction	Human (TSCC tissues), In vitro (CAL-27 cells)	MiR-24 induces invasion and migration of TSCC cells.	[172]

## CircularRNA/lncRNA/miRNA networks and oral cancer metastasis

Noncoding RNAs (ncRNAs) are crucial for most cell biology processes, and also contribute to the growth and spread of malignancies. The ncRNAs that control cancer cell proliferation, cell death, invasion, and metastasis can be divided into circular RNAs (circRNAs), long noncoding RNAs (lncRNAs), as well as miRNAs (discussed above) [236–238]. NcRNAs control the growth of cancer by affecting target gene e asxpression (mRNA) [239]. Both lncRNAs and circRNAs contain binding sites for specific complementary miRNAs, so they can act as competing endogenous RNAs or miRNA sponges, thereby enhancing the expression of the miRNA target genes. If the miRNAs are removed, they can no longer block the translation of mRNAs and silence their target gene expression. The regulatory networks for ceRNAs are associated with the biological basis of cancer [240, 241]. Additionally, circRNAs and lncRNAs may directly interact with various specific proteins to regulate gene transcription [242]. Several studies have examined the role of circRNA-lncRNA-miRNA-gene regulatory networks in oral cancer metastasis, which we summarize below. This research increases our understanding of oral cancer pathogenesis, and could provide new opportunities for less invasive early detection methods and improved therapeutic options.

### LncRNA/miRNA networks

**Fig. 3:**
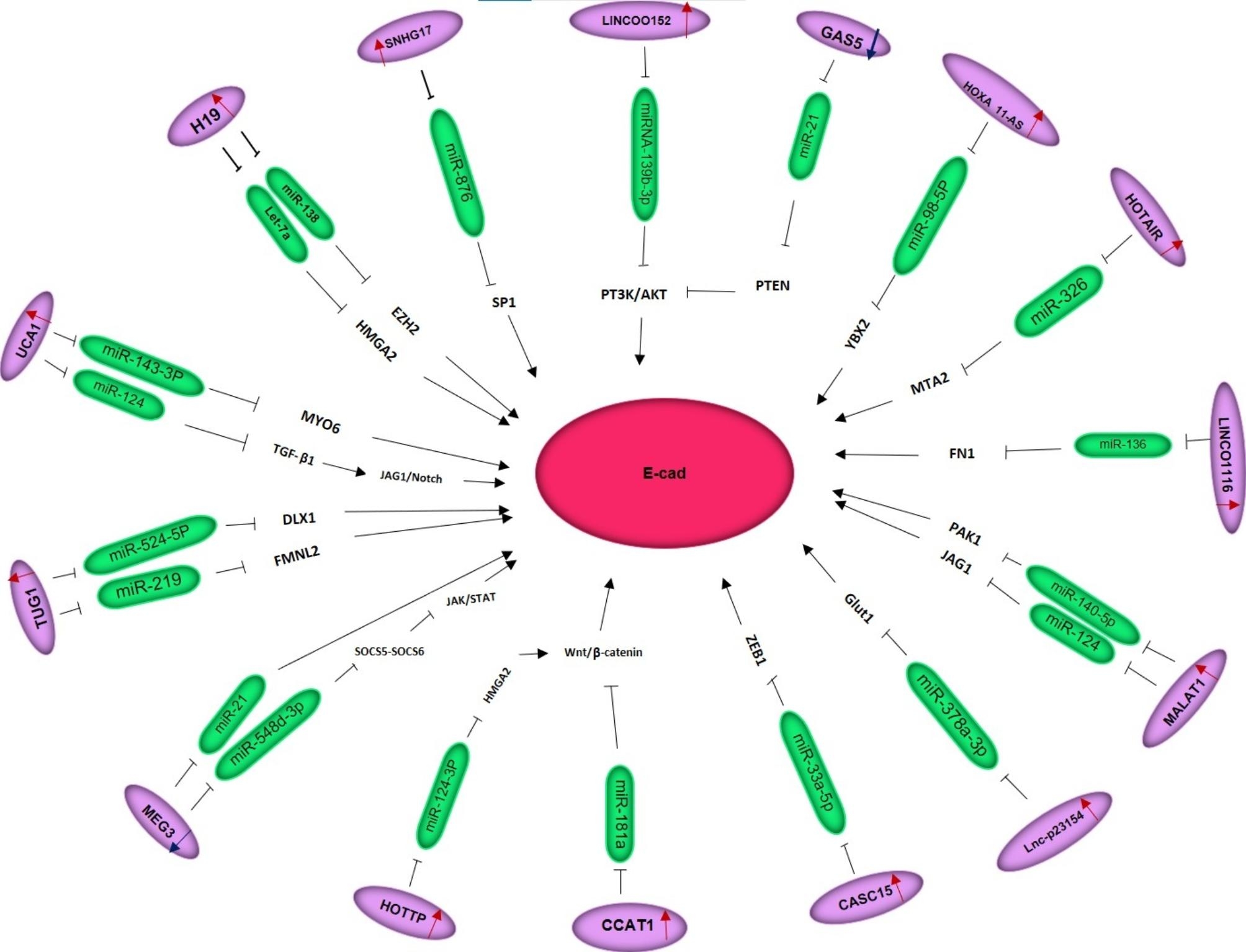
LncRNAs act as competitive endogenous RNAs (ceRNAs). The interactions between lncRNAs and miRNAs play critical roles in the regulation of oral cancer metastasis pathways [285]

LncRNAs are RNA sequences that are over 200 nucleotides long and do not code for any specific proteins. LncRNAs are important for the growth, development, and metastasis of many different cancers, according to accumulating evidence. They are also becoming promising molecular biomarkers for the prognosis and early diagnosis of cancer patients [243, 244]. A variety of tumor types may have lncRNAs intriguing therapeutic targets in and the pathways that they affect (Fig. 3).

A number of human cancers show aberrant expression of LncRNA-H19 [245, 246], which is typically connected to the spread of cancer, a poor prognosis, and cancer recurrence. H19 has also been shown to control the invasion, metastasis, and migration of different malignancies by functioning as a ceRNA [245, 247, 248]. An essential mechanism for how H19 controls OSCC metastasis is its interaction with certain specific miRNAs. Kou et al. [249] discovered that the expression of H19 was higher in metastatic TSCC tissues compared to non-metastatic TSCC tissues, and that H10 knockdown may reduce TSCC cell invasion and migration. According to the proposed mechanism, let-7 was targeted by H19 in order to enhance the expression of HMGA2, a crucial regulator of tumor metastasis. In contrast, TSCC cell migration and invasion were less inhibited by H19 knockdown when let-7a was inhibited [249]. According to a different study, the expression of H19 was inversely connected to overall survival and was favorably associated with the TNM stage. According to another study, H19 could sponge miR-138, thereby increasing the expression of its target gene EZH2, thus promoting OSCC cell invasion, migration, and EMT [32].

LncRNA-MEG3 mostly acts as a tumor suppressor, in contrast to H19. MEG3 is not highly expressed in an all types of cancer according to various studies [250, 251], but when it is expressed it inhibits their invasion, metastasis, and migration. By acting as a ceRNA, it has been shown that MEG3 could also prevent OSCC from migrating and invading. Tan et al. [252], showed that OSCC tissues generally had low MEG3 expression. In return, MEG3 overexpression promoted OSCC cell invasion and migration by downregulation of miR-548d-3p. MiR-548d-3p is inhibited by MEG3, which in turn promotes the production of SOCS5 (cytokine signaling suppressor 5), and SOCS6 (cytokine signaling suppressor 6). Secondly, the JAK/STAT (Janus kinase/signal transducer and activator of transcription) pathway is inhibited by SOCS5/SOCS6 to prevent OSCC invasion and migration [252]. According to a different study, miR-21 and MEG3 were inversely associated in OSCC tissues. The subsequent dual luciferase assay proved that MEG3 and miR-21 directly interacted with each other. Additionally, lowering miR-21 would reduce OSCC cell migration, but inhibiting MEG3 would partially undo the effect of miR-21 downregulation on the migration of OSCC cells [253]. They suggested that MEG3 may sponge miR-21 thereby preventing the OSCC cells from migrating. However, further investigations into the underlying molecular mechanisms are necessary, and confirming the role of MEG3 in OSCC metastasis will require more in vivo experiments.

LncRNAs-TUG1 is an lncRNA which has been intensively studied. TUG1 has been found to enhance the proliferation of OSCC cells by sponging some miRNAs which have an inhibitory effect on the cancer cells. According to Liu et al., OSCC cells showed high TUG1 expression levels which could increase the ability of these cells to migrate. TUG1 and DLX1 (distal-less homeobox 1) may compete with one another for binding to miR-524-5p and thus upregulating DLX1 expression [254]. Additionally, Yan et al. found that TUG1 overexpression boosted OSCC cell capacity to metastasis in vivo while TUG1 knockdown decreased the OSCC cells ability to migrate and invade. Further investigation revealed that TUG1 and miR-219 inhibited one another in a reciprocal fashion, suggesting that suppressing miR-219 in OSCC cells might counteract the inhibitory effects of TUG1 knockdown. They concluded that TUG1 encourages metastasis by functioning as a ceRNA to scavenge miR-219 and increase the expression of the miR-219 target FMNL2 (formin-like protein 2) [255].

LncRNA-UCA1 was first identified in bladder cancer and has since been shown to facilitate bladder cancer cell invasion and migration [256, 257]. Recently, OSCC metastasis has also been linked to aberrant UCA1 expression. UCA1 is overexpressed in TSCC tissues, according to Fang et al., and its degree of expression was strongly linked with lymph node metastasis [258]. Similarly, Zhang et al. found a link between UCA1 overexpression and a poor prognosis in TSCC patients, especially the occurrence of lymph node metastasis and shorter survival. Unexpectedly, UCA1 could sponge miR-124 in TSCC cells and therefore negatively regulate itself. Further research suggested that UCA1 could activate the JAG1/Notch pathway by targeting miR-124 to increase the production of TGF-1, thus promoting TSCC cell invasion and EMT [259]. Additionally, in a different study, it was found that UCA1 competitively bound to miR-143-3p and affected MYO6 (myosin VI) expression thus enhancing TSCC cell invasion, EMT and migration [260].

### CircRNA/miRNA networks

**Fig. 4:**
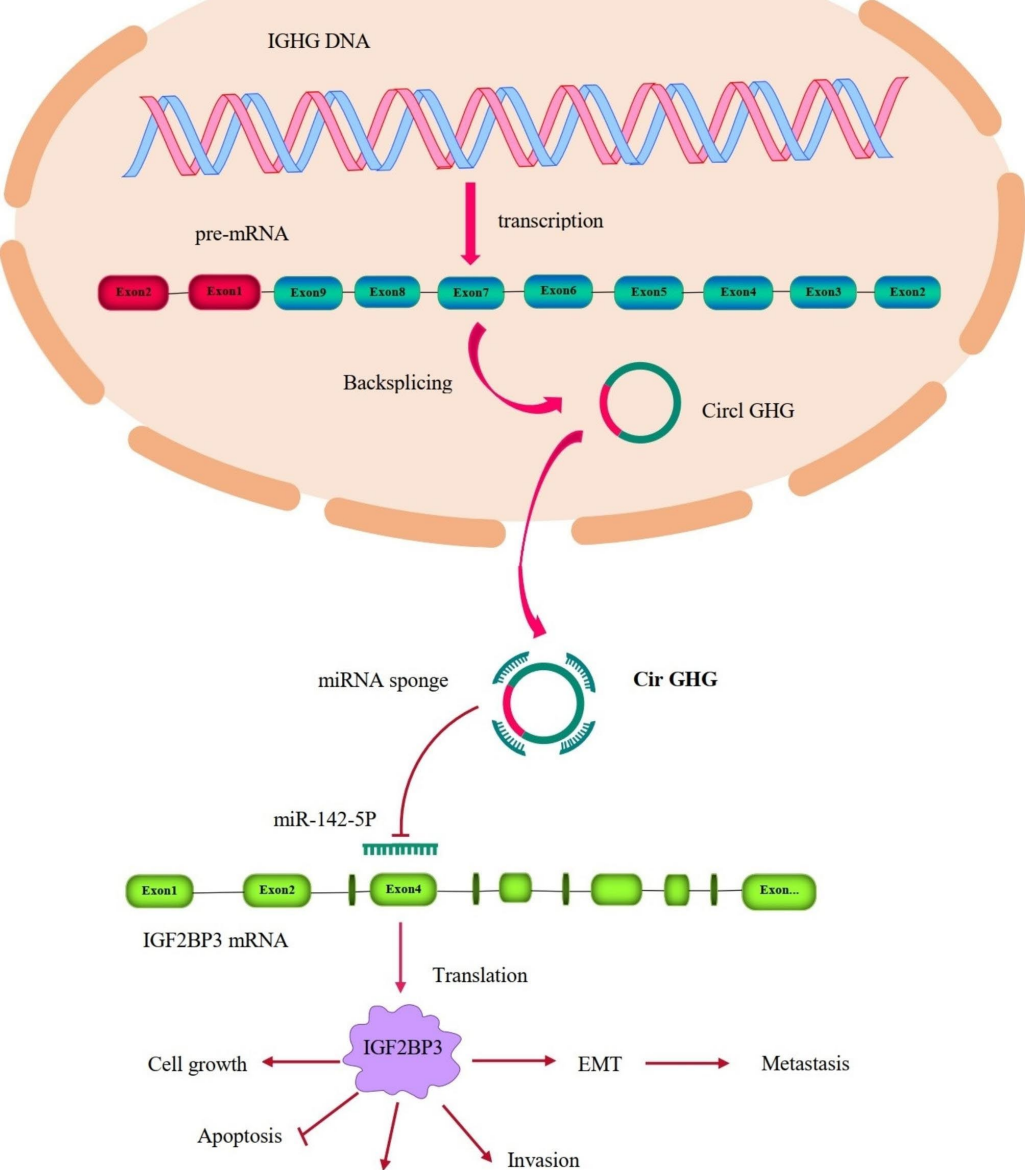
Interaction between circIGHG and miR-142- 5p/IGF2BP3 in OSCC. CircIGHG is a circRNA derived from the IGHG locus. The expression of circIGHG is increased in OSCC cells and is positively correlated with poor prognosis in OSCC. MiR-142-5p can inhibit OSCC metastasis by targeting IGF2BP3, in return, circIGHG promotes EMT activation of OSCC cells by sponging miR-142-5p and upregulating IGF2BP3 [275]

CircRNAs are a group of closed circular single-stranded RNA molecules that can regulate gene expression at both the transcriptional and posttranscriptional levels [261]. In 2012, Salzman et al. [262] reported that more than 10% of expressed genes have the potential to create circRNAs. Circular transcripts of the protein linked to cerebellar degeneration 1 (CDR1, also called ciRS-7) antisense RNA were discovered to act as miR-7 sponges, according to a 2013 study by Hansen et al., and Memczak et al. [263, 264]. These studies made circRNAs a new focus for scientific investigation in the noncoding RNA field. CircRNAs significantly contribute to signaling networks that promote the growth and spread of cancer. For example, lncRNA-WDFY3-AS2 suppressed OSCC cell metastasis by targeting the Wnt/β-catenin signaling pathway [265]. The migration, invasion, and metastasis of OSCC are likewise affected by dysregulated circRNA expression since these genes are regulated by sponging of various miRNAs (Table 3; Fig. 4) For instance, Xia et al. reported that circ-0001162 (circ-MMP9), a metastasis-associated circRNA, was elevated in OSCC samples [266]. Given its strong relationship with MMP9 expression, circ-MMP9 may serve as a sponge for miR-149 to target AUF1. This was shown to be correct both in vitro and in vivo. Circ-MMP could prevent OSCC from spreading by inhibiting MMP9 expression. CircUHRF1 may up-regulate c-Myc by acting as a miR-526b-5p sponge, which may enhance the transcription of ESRP1 and TGF-1. In OSCC, CircUHRF1 was markedly overexpressed [267]. Additionally, it was found that circUHRF1 could circularize and increase with the aid of ESRP1, creating a feedback loop between those two factors, as well as TGF-1, c-Myc, miR-526b-5p, and ESRP1 that working together could promote OSCC carcinogenesis and EMT [267]. Studies conducted in vitro have shown that inhibition of circUHRF1 could reduce the ability of OSCC cells to migrate, proliferate, invade, and undergo the EMT. Additionally, in vivo functional tests demonstrated that blocking circUHRF1 could effectively halt OSCC tumor growth. When circ-PKD2 was overexpressed the ability of miR-204 to promote cancer metastasis was dramatically reduced, because OSCC migration and and invasion was inhibited. An in vivo study showed that the size and weight of OSCC xenografted tumors were greatly reduced by overexpression of circ-PKD2 [267]. Dual-luciferase reporter analysis confirmed that miR-204-3p and circ-PKD2 directly interacted, and miR-204-3p targeted APC2 through a downstream signaling pathway [22]. The extracellular signal-regulated kinase 1/2, protein kinase B, and β-catenin pathways were all inhibited by circ-PKD2 because it up regulated APC2 and reduced the inhibitory effect of miR-204-3p. Moreover another circRNA called circDOCK1 was found to strongly expressed in OSCC cells, and it was discovered that circDOCK1 targeted miR-196a-5p [22].

Circ-0000140 showed markedly decreased expression in OSCC patient samples [268]. According to one study, low circ-0000140 expression might function as a biomarker for OSCC progression, because it is strongly associated with OSCC patient poor prognosis [23, 269]. However, further research into the function and mechanism of circ-0000140 in OSCC is needed. Recently, Peng et al. [269] reported that OSCC patients showed downregulation of circ 0000140, and low circ-0000140 expression was correlated with lymph node metastasis and more advanced TMN stage. Additionally, survival analysis revealed that OSCC patients with low circ-0000140 expression levels had a significantly worse 5-year survival rate. Mechanistic studies in vitro showed that OSCC cell proliferation, invasion and migration were all inhibited by the overexpression of circ-0000140. Further evidence that circ0000140 inhibited the EMT in OSCC cells was provided by the higher E-cadherin and lower N-cadherin protein levels observed in OSCC cells with overexpressed circ-0000140 [269]. When circ0000140 was overexpressed in a xenograft mouse model, in addition to shrinking the tumor, it also prevented lung metastasis of tumors grown from two separate OSCC cancer cell lines. Moreover, it was shown that overexpression of circ-0000140 caused a > 50% reduction in metastatic lung nodules in an in vivo model. Furthermore, circ-0000140 was found to bind to miR-31 and thus increase the expression of its target gene LATS2, which would have an effect on the EMT in OSCC cells [269]. LATS1/2 are significant regulators of both tumor-suppressor as well as oncogenic effectors during cancer spread [270]. Taken together, these findings showed that circ-0000140 inhibited OSCC metastasis via targeting the miR-31/LATS2 axis [269]. In accordance with the findings of Peng et al. [269], Guo and colleagues [23] found that OSCC showed low levels of circ-0000140 expression, and its overexpression prevented cell invasion and migration, and reduced glycolysis. When circ-0000140 was overexpressed it suppressed OSCC tumor growth in vivo. Moreover, they found that circ-0000140 sponged miR-182-5p which targeted CDC73. A miR-182-5p mimic reversed the inhibition of OSCC caused by circ 0000140 overexpression. They discovered that circ-0000140 may prevent OSCC from spreading by controlling the miR-182-5p/CDC73 axis [23].

Recently, Hei and colleagues found that the expression level of circ_0020377 was significantly upregulated in OSCC tissue samples and OSCC cell lines. Also, they found that miR-194-5p acted as tumor suppressor miRNA in OSSC cells by targeting KLF7. Furthermore, they observed that upregulated circ_0020377 promoted OSCC cancer development and metastasis. Mechanistically, they suggested that circ_0020377 promoted invasion and migration of OSCC cells by by sponging miR-194-5p and thus upregulating KLF7 [271]. These findings suggest that further investigation is required into the circRNA-miRNA networks which control signaling pathways and related genes that are relevant to the development and spread of oral cancer.

**Table 3:** LncRNA/circRNA/miRNA networks in oral cancer metastasis

Oral cancer type	circRNA/ lncRNA (expression)	miRNA (Expression)	Target of miRNA	Inhibition/ Induction of metastasis by miRNA	Results	Ref
OSCC	circVAPA (Up)	miR-132 (Up)	HOXA7	Inhibition	CircVAPA promoted OSCC growth by sponging miR-132, which likely suppressed the invasion, migration, and metastasis of oral cancer cells by targeting HOXA7.	[272]
OSCC	LINC00662 (Up)	miR-144-3p (Down)	EZH2	Inhibition	LINC00662 caused EMT, migration, and invasion of OSCC by sponging miR-144-3p.	[25, 26]
OSCC	LINC00319 (Up)	miR-199a-5p (Down)	FZD4	Inhibition	LINC00319 promoted OSCC metastasis by sponging miR-199a-5p.	[273]
OSCC	ZEB1-AS1 (Up)	miR-23a (Down)	-	-	Lnc-ZEB1-AS1 induced OSCC metastasis by decreasing the expression of miR-23a.	[274]
OSCC	circIGHG (Up)	miR-142-5p (Down)	IGF2BP3	Inhibition	MiR-142-5p prevented OSCC metastasis possibly by targeting IGF2BP; conversely, circIGHG promoted EMT of OSCC cells by sponging miR-142-5p.	[275]
TSCC	LTSCCAT (Up)	miR-103a-2-5p (Down)	SMYD3	Inhibition	MiR-103a-2-5p prevented TSCC cells from metastasizing by targeting SMYD3, whereas lnc-LTSCCAT promoted TSCC metastasis.	[276]
Oral cancer stem cell	Lnc-MEG3 (Up)	miR-421 (Down)	-	Inhibition	Lnc-MEG3 promoted the ability of oral cancer stem cells to invade, but co-transfection with miR-421 mimics suppressed the effect of lnc-MEG3.	[277]
TSCC	Lnc-ADAMTS9-AS2 (Up)	miR-600 (Down)	EZH2	Inhibition	Lnc-ADAMTS9-AS2 promoted TSCC migration and EMT by targeting miR-600.	[278]
OSCC	circDHTKD1 (Up)	miR-326 (Down)	GAB1	Inhibition	miR-326 may inhibit OSCC metastasis by downregulating GAB1 expression, conversely, circDHTKD1 induced metastasis of OSCC by sponging miR-326.	[279]
OSCC	circ_0020377	miR-194-5p	KLF7	Inhibition	circ_0020377 promoted invasion and migration of OSCC cells by upregulating KLF7 expression levels through sponging of miR-194-5p.	[271]
OSCC	lnc- SNHG5 (Up)	miR‑655‑3p (Down)	FZD4	Inhibition	Lnc-SNHG5 promoted OSCC invasion and migration by sponging miR655-3p. MiR-655-3p targeted FZD4 and competed with lnc-SNHG5 to suppress the invasion and migration of SCC-4 cells.	[280]
OSCC	lnc-RC3H2 (Up)	miR-101-3p	EZH2	Inhibition	Lnc-RC3H2 promoted migration and invasion of OSCC cells by sponging miR-101-3p, . Upregulation of miR-101-3p targeted EZH2 to reduce RC3H2-induced invasion and migration in OSCC.	[281]
OSCC	linc01234 (Up)	miR-433-3p	PAK4	Inhibition	Linc01234 targeted miR-433-3p to promote OSCC metastasis	[282]
OSCC	lnc-TIRY (Up)	miR-14 (Down)	-	Inhibition	Lnc-TIRY activated the Wnt/β-catenin pathway and promoted OSCC metastasis by sponging miR-14.	[283]
OSCC	Lnc-GAS5	miR-21	PTEN	Induction	Lnc-GAS5 inhibited invasion, migration, and EMT of OSCC cells by targeting miR-21/PTEN axis.	[24]
OSCC	circ_0000140 (Down)	miR-31 (Up)	LATS2	Induction	Circ_0000140 blocked OSCC invasion and migration by sponging miR-31, which in turn blocked the Hippo signaling pathway. On the other hand, miR-31 encouraged OSCC cell invasion and migration by inhibiting LATS2.	[284]
OSCC	circ_0000140 (Down)	miR-182-5p (Up)	CDC73	Induction	Circ_0000140 inhibited metastasis of OSCC by increasing CDC73 through targeting miR-182-5p.	[23]
OSCC	circ-PKD2 (Down)	miR-204‐3p (Up)	APC2	Induction	Upregulation of miR-204‐3p promoted metastasis by increasing invasion and migration. CircPKD2 reduced OSCC migration and invasion by targeting miR204-3p	[22]

## Conclusion

Metastatic cancer is still mostly incurable due to the lack of effective clinical treatment and our limited understanding of how oral cancer metastasizes. Our understanding of the roles of certain miRNAs in metastasis has advanced a lot in the last ten years. In order to treat oral cancer, many of these metastasis-inducing miRNAs could be attractive therapeutic targets. It should be emphasized that certain lncRNAs or circRNAs have the ability to regulate the actions of specific miRNAs. Moreover, a single miRNA (or a miRNA cluster/family) may play more than one role in the invasion-metastasis cascade. For example, miR-200 family members inhibit tumor cell EMT, migration, and infiltration, but they can also aid in metastasis by increasing the suitability of the OSCC microenvironment for invasion. Therefore, prior to entering clinical trials, the therapeutic potential of miRNA-based therapeutics should be rigorously assessed using relevant laboratory models and in real life clinical situations. A full understanding of the role of miRNA biogenesis and their mode of action in metastasis requires further study. Crosstalk between miRNAs and lncRNAs/circRNAs has recently become a fascinating study area (Table 3). Nonetheless, functional investigations have shown that miRNAs are an important pathway by which ceRNAs can control oncogenes and tumor suppressor genes. Therefore, future studies must pay greater attention to the circRNA/lncRNA-miRNA-mRNA networks in oral cancer metastasis.

## Data Availability

Not applicable.
